# Inverted laser sintering of metal powders

**DOI:** 10.1038/s41598-023-47184-8

**Published:** 2023-11-16

**Authors:** John Whitehead, Hod Lipson

**Affiliations:** Department of Mechanical Engineering, Columbia University, New York, USA

**Keywords:** Mechanical engineering, Design, synthesis and processing

## Abstract

We demonstrate the ability of the inverted laser sintering process to manufacture parts composed of metal powder. We fabricate a 10-layer part by depositing a layer of copper powder onto a sapphire plate, then pressing the plate against the part being built and sintering the powder onto the part by shining a 14W 445 nm laser through the glass. The process was then repeated multiple times, each time adding a new layer to the component being printed until completion. We discuss the potential applications and impacts of this process, including the ability to directly fabricate multi-material metallic parts without the use of a powder bed.

## Introduction

As the industrial application of Additive Manufacturing technologies continues to grow at an annual rate of 19.5% percent^1^, the majority of that growth is in the area of metal laser sintering or melting. Laser sintering involves selectively sintering metallic particles in a powder bed, according to an electronic blueprint^2–4^. A key challenge to the use of powder beds however is the many practical as well as safety and environmental constraints they introduce. From a practical point of view, powder beds are cumbersome to operate, fill and clean. Additionally, handling of powder can be unsafe and environmentally hazardous^5,6^.

Metal parts are traditionally additively manufactured using either selective laser sintering (SLS), selective laser melting (SLM), or binder Jetting (3DP)^7^. These processes work by first placing down a thin layer of powder onto a print bed. A laser or binder jet is then directed down into this powder layer, selectively fusing more melting a region of powder particles together. The print bed then moves down, and another layer of powder is deposited onto the previous layer. The laser again fuses a region of powder in this new layer, which also fuses this new layer to the previous layer. This process is then repeated until a solid object is formed. The solid object is excavated from the powder bed in which it is buried, dusted off, cleaned and finished, while removing any auxiliary support structure^8–10^. Additional annealing is sometimes applied. Some of the unused powder in the bed can be recycled. Other methods involving wire feed, extrusion, or powder jetting are also used for additive manufacturing of metal parts^11–13^, though they are less commonly used.

Here we report on a process that can sidestep the use of the powder bed entirely, while maintaining the use of powders. This process begins by coating the upper surface of a sapphire glass plate with a thin layer chosen powder. The powder can be dispensed from a “salt shaker”, ground off a solid stock material, or lifted from a powder vat using electrostatic charge. The glass pressed onto the part, and a laser beam is directed through the glass. The laser beam is directed to selectively fuse a desired region of the powder onto the substrate. The substrate can then be lifted off the part, the material on the glass can be replenished, and the process can be repeated, with material in the new powder layer being fused to material in the previous layer.

Since this process stacks successive layers of material without using a powder bed, the printed part is fully exposed and visible during the entire process. Therefore, unlike traditional SLA and FDM printers, failure can easily be detected in real time and addressed or aborted, eliminating wasted time and materials^14,15^.

**Figure 1:**
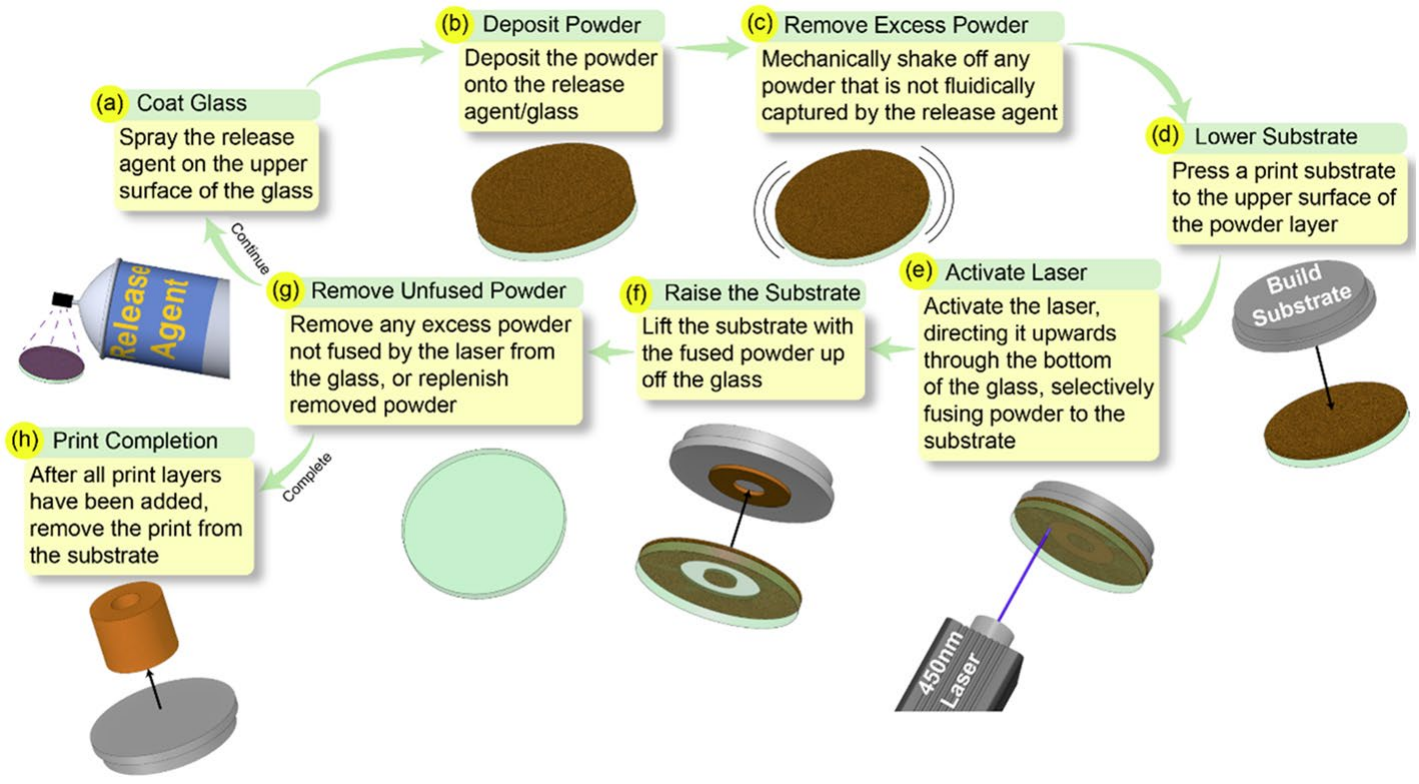
(**a**) Coat the glass with the release agent (**b**) Deposit material powder onto the release agent (**c**) Remove powder not captured by release agent (**d**) Press a substrate to the upper surface of the powder layer (**e**) Direct the laser through the glass in a pre-programmed pattern (**f**) Raise the substrate with the fused material powder (**g**) Remove unfused powder still on the glass h) Repeat process until the print is completed, remove the print from the substrate.

In previous work^16–18^, we showed how this process could be used to additively manufacture polymer parts (Fig. 1). A key challenge has been to apply this process to metals, rather than polymers. The application to metals is not straightforward, owing to the tradeoff between the high temperature of metal sintering and the thermal sensitivity of the glass plate. Here we report on the first successful implementation of this concept.

## Materials and methods

Our testing setup required a laser that could transmit through a clear glass surface and would be able to process metal powders. For this manufacturing process, we utilized a blue 445 nm diode laser. This laser operated at ~ 14W (J Tech Photonics, LD445_14W_Head). For the metal powder, we selected copper (Sigma-Aldrich, < 75 μm particle size, 207,780)^19,20^ because it can more effectively absorb blue light when compared to alternative metal powders.

The laser was kept at a fixed position and directed upwards, and the material coated glass was moved above it using a gantry controlled using a Smoothieboard 5X. The glass selected for these experiments was 1 mm thick round sapphire plate (Al_2_O_3_, Edmund Optics). This glass was chosen as it allows the blue light lasers to pass through with minimal absorption while also being robust enough to endure contact with the laser processed metal powder^21,22^. Alternative glass material tested were quartz and borosilicate, which also promised good transmissivity in the blue wavelength range^23,24^. However, all these samples blemished or cracked under the use of a laser.

To ensure a thin layer of copper powder was deposited onto the glass, we utilized a coating method that we previously demonstrated in the inverted polymer sintering processes. This method utilizes a release agent (Water Soluble Mold Release Agent, SLIDE) which was sprayed onto the upper surface of the glass. The powder was then deposited onto the top of this release agent layer. The liquid nature of this spray allowed it to capture a single layer of powder, with excess uncaptured powder being shaken off. Alternative methods for loading the powder, such as through electric charge, could also be used.

For these experiments, substrates were fabricated from Tin (Sn) due to its favorable binding properties. The substrate was thoroughly sanded to remove surface impurities, then mounted onto a rubber spacer to insulate the tin from the surrounding air and increase heat retention in the substrate. The relatively low melting temperature of tin when compared to copper helped propagate adhesion of the initial copper layers to the moveable substrate.

**Figure 2:**
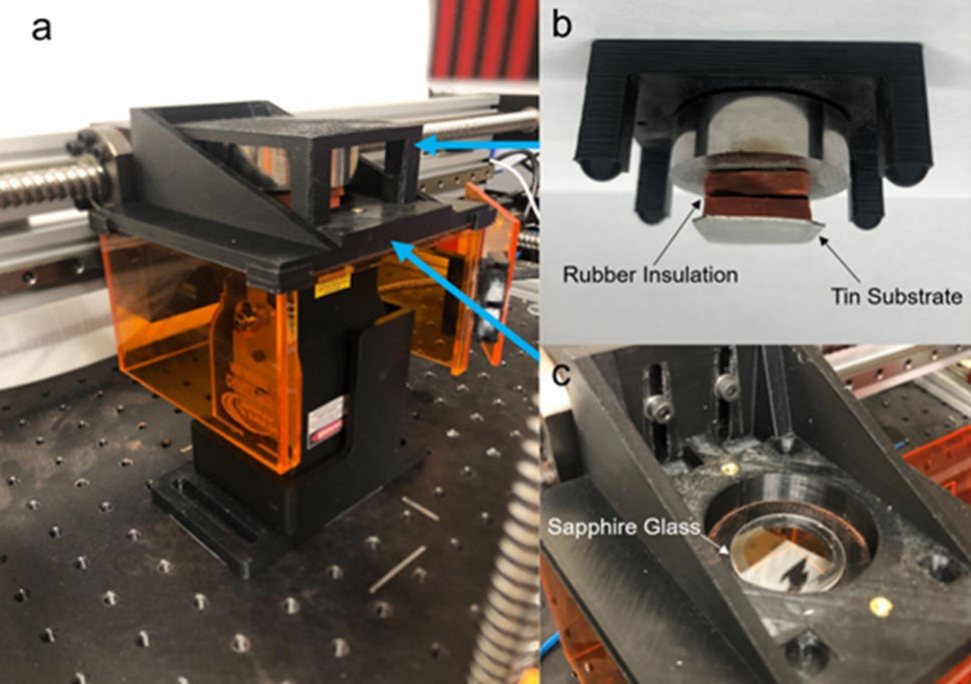
(**a**) Testing setup with the glass holder/substrate mounted to an XY gantry above the laser, orange acrylic shielding is used to block any errant laser light (**b**) The tin substrate attached to rubber insulation sheeting, mounted to the upper platform (**c**) Image of the uncoated sapphire glass in the holder mounted over the laser.

For demonstration, we attempted to produce a multi-layer copper sample. This was accomplished by first lowering the substrate onto the powder coated glass. During the printing process, the substrate/rubber was mounted to a moveable platform that can be raised and lowered above the laser/glass. The glass was placed onto a holder, which was mounted to an XY gantry (Fig. 2). This XY gantry moved the glass/substrate above the laser. During the laser cycle, bolts held the platform/substrate firmly against the powder coated glass to ensure consistent contact between the substrate and the powder. These bolts can be seen in supplementary video 1, which shows a single layer test print. Due to the manual nature of the bolt tightening, the applied substrate load varied from layer to layer. However, the loads applied were still sufficient to ensure solid contact across the print/powder layer interface, allowing for new layers to be added to the previously printed ones.

The glass/substrate assembly (GSA) was moved in a concentric/nested circle pattern above the laser to fuse the powder, starting with the innermost circle. The gantry motion settings were kept consistent throughout the entire printing process. Each circle was comprised of 360 points, and the GSA moves the laser between them at a straight-line speed of 400 mm/min. The circle radius is then increased by 0.1 mm, and the process is repeated until a preselected outer radius is reached. The laser cycle is then repeated, so each nested circle is processed twice. Speed settings were kept consistent throughout the entire printing process and were determined experimentally. Once the laser cycles were completed, the substrate was raised with the fused material, the glass recoated, before the process repeated until a multi-layer part was generated.

## Results and discussion

**Figure 3:**
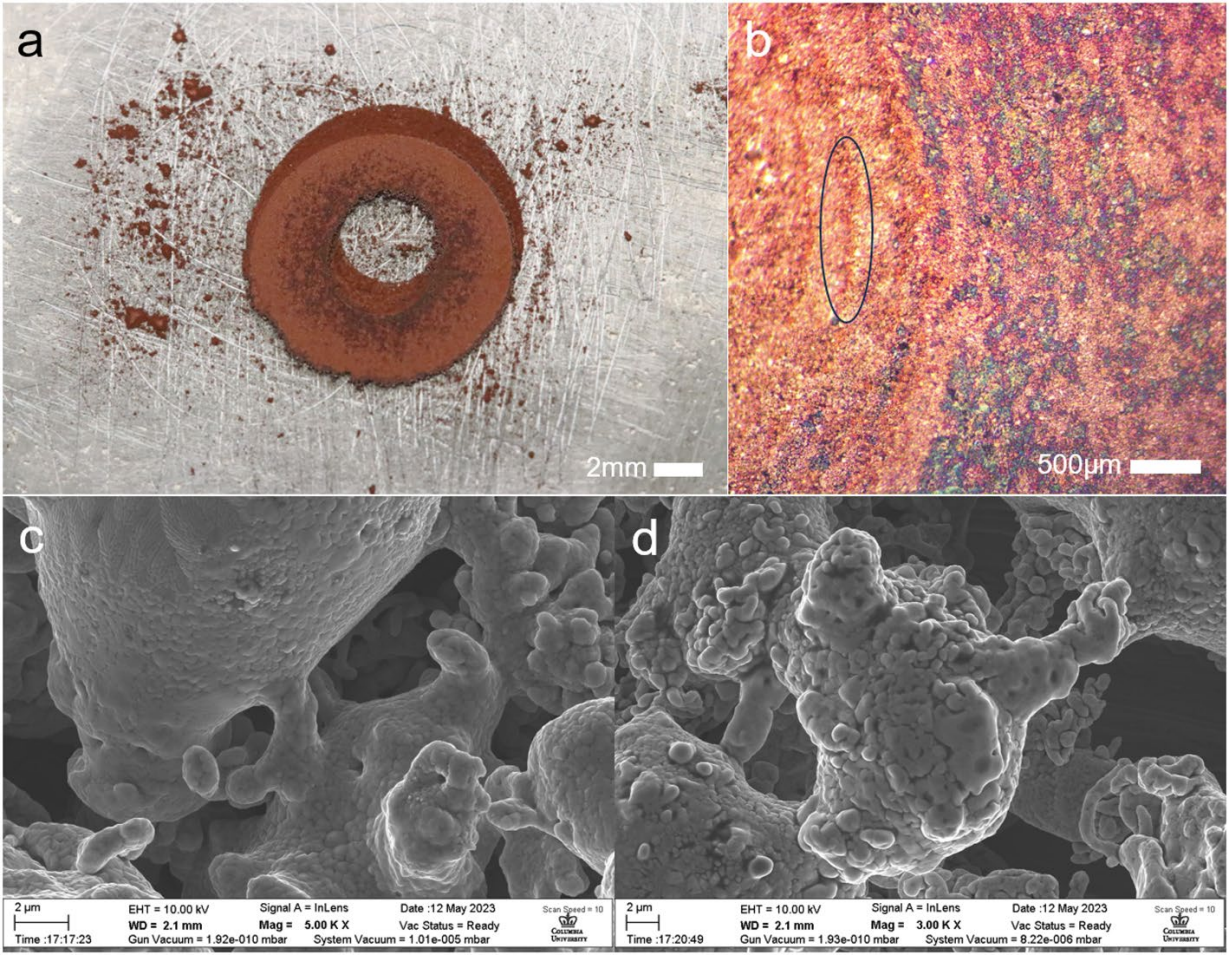
A 10-layer copper washer printed using the proposed process. (**a**) Multi-layer copper print sample (**b**) Magnified view of the upper print surface, towards the left side of the image layer boundaries can be seen, one is highlighted with the black oval (**c**, **d**) SEM observations of necking behavior in different regions of the copper powder processed print layer.

We produced a print sample composed of copper powder (Fig. 3a). This was successfully done without damaging the glass to any extent visible to the naked eye. This print was comprised of 10 layers. Due to the manual way in which the platform was raised and lowered during the printing process, layer-to-layer alignment was imperfect, which can be seen when looking at the print (Fig. 3b), as it is possible to see regions of previous layers past the surface of the “final” layer.

As these experiments are done in open air, oxidation of the processed metal was highly likely, and can clearly be seen in some of the darkened regions of the print. This oxidation reduces the strength of the print sample, and any practical implementation of this printing process would need to be done in a de-oxygenated environment.

In addition to the 10-layer print, we fabricated single layer samples to observe in-layer behavior. For analysis, a ZEISS sigma VP scanning electron microscope was used to analyze powder bonding behaviors. Particles seem to generally exhibit early-stage necking behaviors, although some areas with greater oxidation appear to have undergone more melting (Fig. 3c, d). The minimal extent to which the powder was bonded implies the need for some form of post processing to better fuse the particles, either utilizing bulk sintering or a secondary laser.

## Conclusion, limitations, and future work

We have shown that it is possible to fabricate copper part using an inverted laser sintering setup. At present, particle bonding is relatively minimal, due to the relatively lower power of the laser. Post processing methods could be used to increase bonding, such as bulk sintering.

Additionally, in previous experiments, we have demonstrated the ability of this process to print polymer materials. The addition of copper opens the door to prints containing both metals and plastics. This would allow for production of things like multi-layer circuit boards, produced using the proposed process. The metal regions would here act as conductive channels, not as structural support.

Due to the minimal level of sintering, in this paper we did not have to compensate for any layer thickness change caused by heating. However, future project iterations that seek to cause greater sintering/melting during the laser stage will need to take change in layer thickness into account.

Future work will include developing methods to generate support structures to allow for more complex prints.

### Supplementary Information

Supplementary Video 1.

Supplementary Information 1.

## Data Availability

All data generated or analyzed during this study are included in this published article [and its supplementary information files].
