# Supplementary material for: Inverted laser sintering of metal powders
Source: Sci Rep. 2023 Nov 16;13:20013. doi: 10.1038/s41598-023-47184-8 (PMC10654679; doi:10.1038/s41598-023-47184-8)
Supplement: Supplementary file 2 — Supplementary Information 1. [file 41598_2023_47184_MOESM2_ESM.docx]

Supplementary Video 1: The glass is moved above the laser with the substrate pressed to it. After the laser cycle is complete, the substrate is raised, and it can be seen that a select region of powder was fused to the tin.
